# Pre-Excited Atrial Fibrillation in Wolff-Parkinson-White (WPW) Syndrome: A Case Report and a Review of the Literature

**DOI:** 10.31083/j.rcm2504125

**Published:** 2024-03-29

**Authors:** Marco Schiavone, Annalisa Filtz, Alessio Gasperetti, Xiaodong Zhang, Giovanni B. Forleo, Pasquale Santangeli, Luigi Di Biase

**Affiliations:** ^1^Department of Clinical Electrophysiology & Cardiac Pacing, Centro Cardiologico Monzino, IRCCS, 20138 Milan, Italy; ^2^Department of Systems Medicine, University of Rome Tor Vergata, 00133 Rome, Italy; ^3^Cardiology Unit, Luigi Sacco University Hospital, 20157 Milan, Italy; ^4^Department of Medicine, Division of Cardiology, Johns Hopkins School of Medicine, Baltimore, MD 21218, USA; ^5^Cardiac Arrhythmia Center, Division of Cardiology at Montefiore-Einstein Center, Bronx, NY 10467, USA; ^6^Department of Cardiovascular Medicine, Cleveland Clinic, Cleveland, OH 44106, USA

**Keywords:** pre-excited atrial fibrillation, Wolff-Parkinson-White syndrome, accessory pathway, antiarrhythmic drugs, catheter ablation

## Abstract

Wolff-Parkinson-White (WPW) syndrome is defined by specific electrocardiogram (ECG) changes resulting in ventricular pre-excitation (the so-called WPW pattern), related to the presence of an accessory pathway (AP), combined with recurrent tachyarrhythmias. WPW syndrome is characterized by different supraventricular tachyarrhythmias (SVT), including atrioventricular re-entry tachycardia (AVRT) and atrial fibrillation (AF) with rapid ventricular response, with AVRT being the most common arrhythmia associated with WPW, and AF occurring in up to 50% of patients with WPW. Several mechanisms might be responsible for AF development in the WPW syndrome, and a proper electrocardiographic interpretation is of pivotal importance since misdiagnosing pre-excited AF could lead to the administration of incorrect treatment, potentially inducing ventricular fibrillation (VF). Great awareness of pre-excited AF’s common ECG characteristics as well as associated causes and its treatment is needed to increase diagnostic performance and improve patients’ outcomes. In the present review, starting from a paradigmatic case, we discuss the characteristics of pre-excited AF in the emergency department and its management, focusing on the most common ECG abnormalities, pharmacological and invasive treatment of this rhythm disorder.

## 1. Introduction

Wolff-Parkinson-White (WPW) syndrome was first described in the 1930s, being at that time associated with sudden cardiac death (SCD). If most patients with WPW syndrome are asymptomatic, symptomatic cases may range from sporadic palpitations to recurrent supraventricular tachycardia (SVT) resulting in syncope, hemodynamic instability and SCD. Patients with hyperthyroidism are generally more prone to develop cardiac arrhythmias, and especially atrial fibrillation (AF), being very difficult to manage in this scenario. This event is particularly detrimental in this setting, since the faster conduction of the accessory pathway (AP) during pre-excited AF, when compared to the atrioventricular node, may induce ventricular fibrillation (VF). Hereby we report a case of pre-excited AF during a thyroid storm, along with an in-depth review of the literature.

## 2. Case Report

**Fig. 1.**
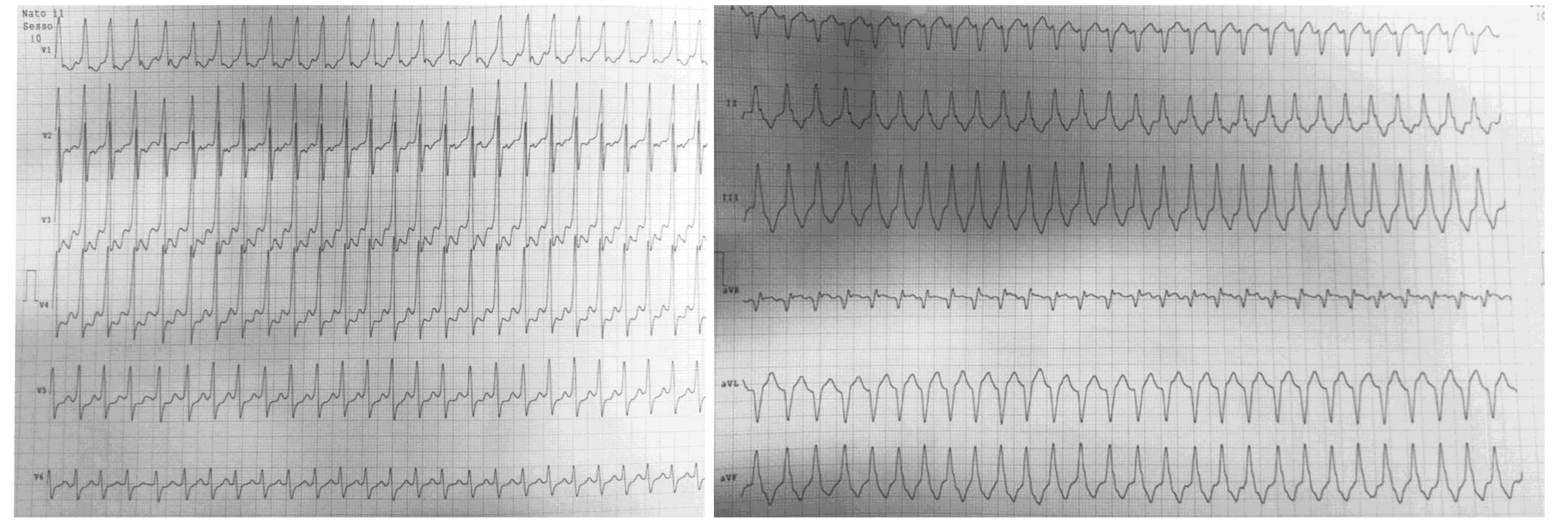
**The first collected electrocardiogram (ECG) at the emergency department admission depicted a mildly wide complex tachycardia with a slurred QRS upstroke and slightly irregular cycle length**. The initial differential diagnosis included antidromic atrioventricular re-entry tachycardia (AVRT), atypical AV node re-entry tachycardia (AVNRT) with a bystander accessory pathway (RP not compatible with typical AVNRT), atrial tachycardia (AT) or atrial flutter conducted through an accessory pathway (possibly with 2:1 conduction), or theoretically any supraventricular tachycardia (SVT) with aberrancy due to phase-3 bundle branch block.

**Fig. 2.**
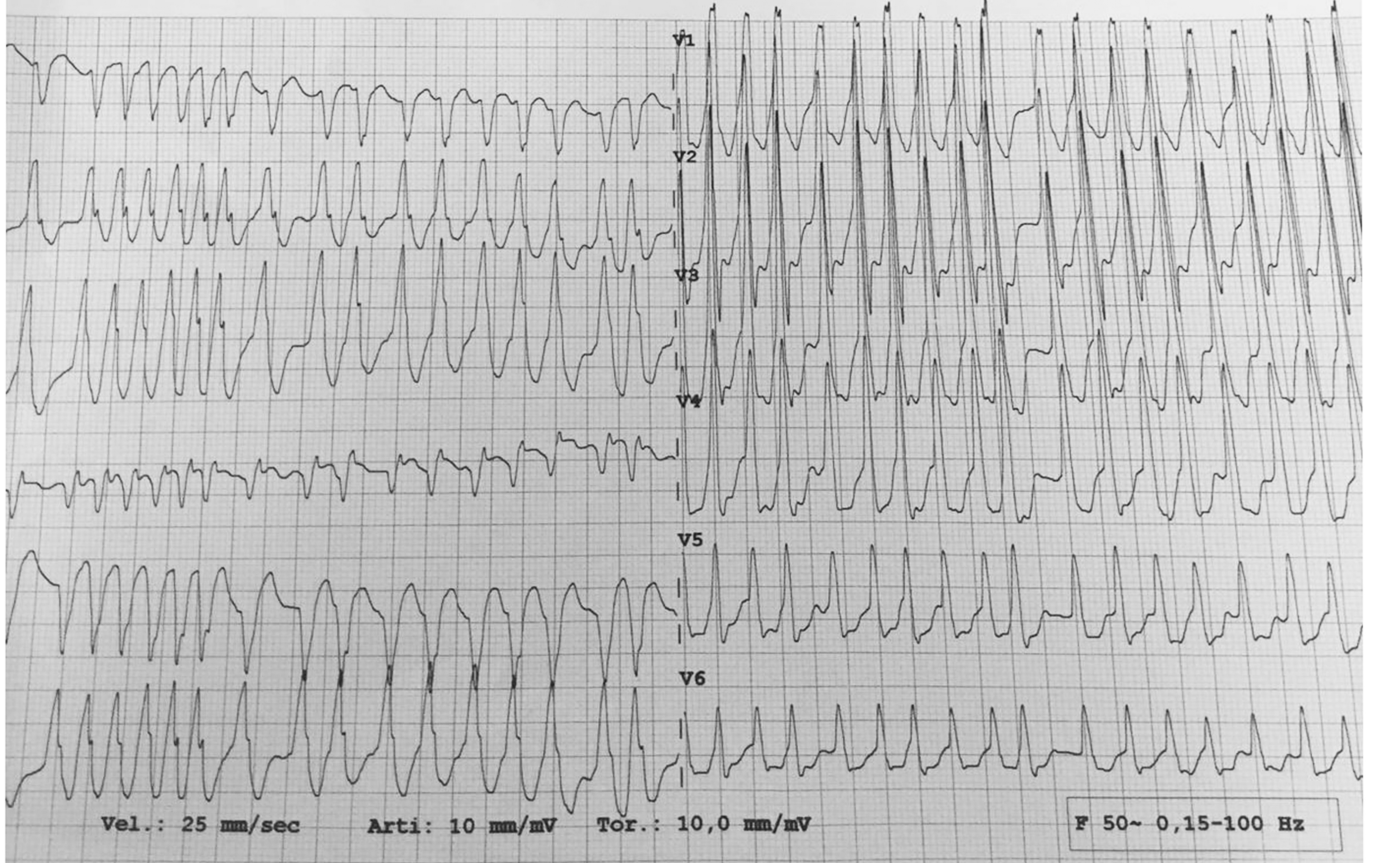
**The ECG monitoring revealed phases of irregular tachycardia cycle lengths, thereby confirming pre-excited atrial fibrillation**. Utilizing algorithms capable of predicting accessory pathway (AP) location, the ECG suggested the presence of a left lateral AP. Identifying the correct location is best achieved when there is maximal pre-excitation, as observed in this tracing. ECG, electrocardiogram.

**Fig. 3.**
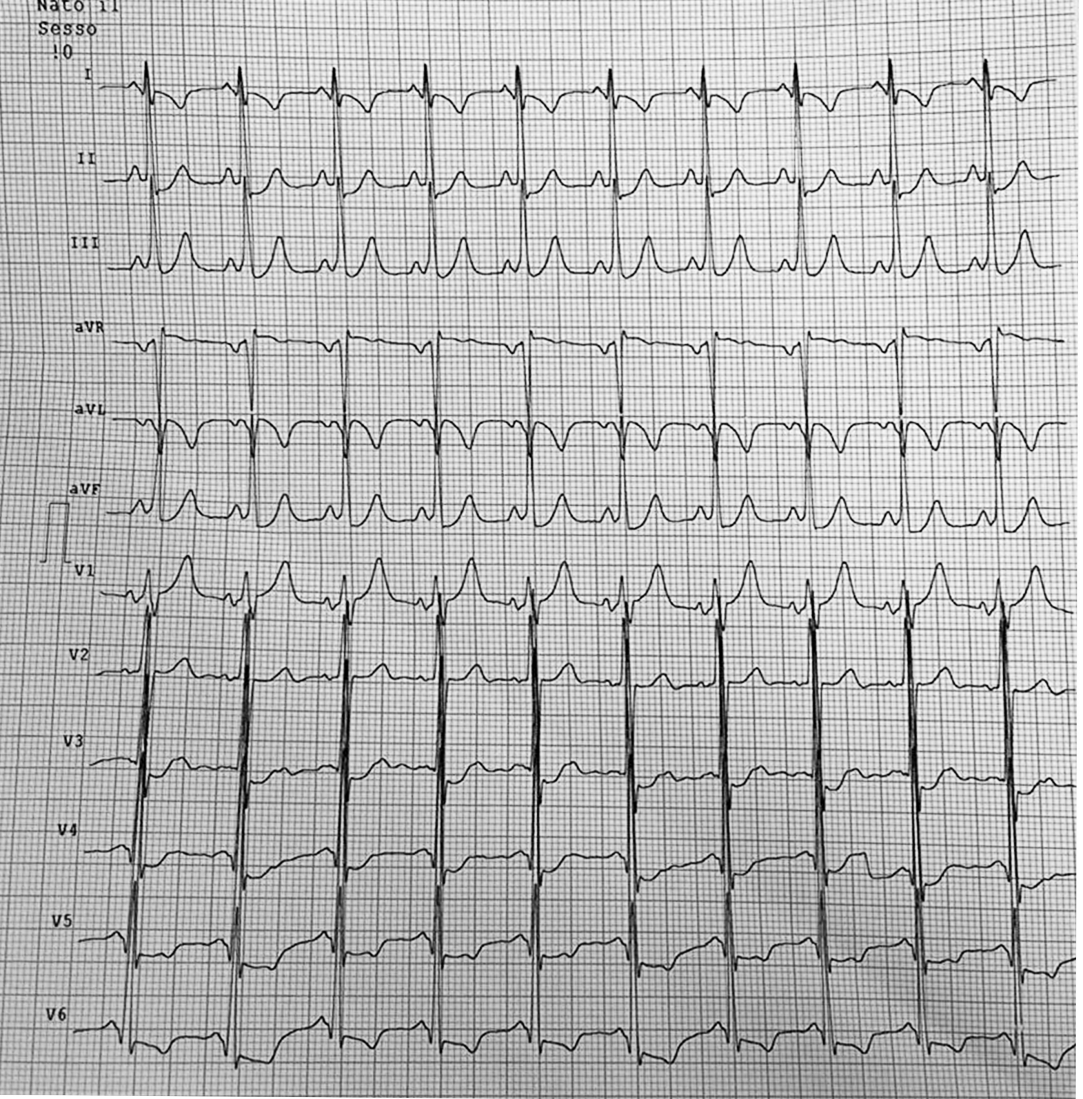
**The ECG during sinus rhythm displayed a short PR interval (90 msec) and a mild delta wave, which was more pronounced in the inferior leads**. ECG, electrocardiogram.

**Fig. 4.**
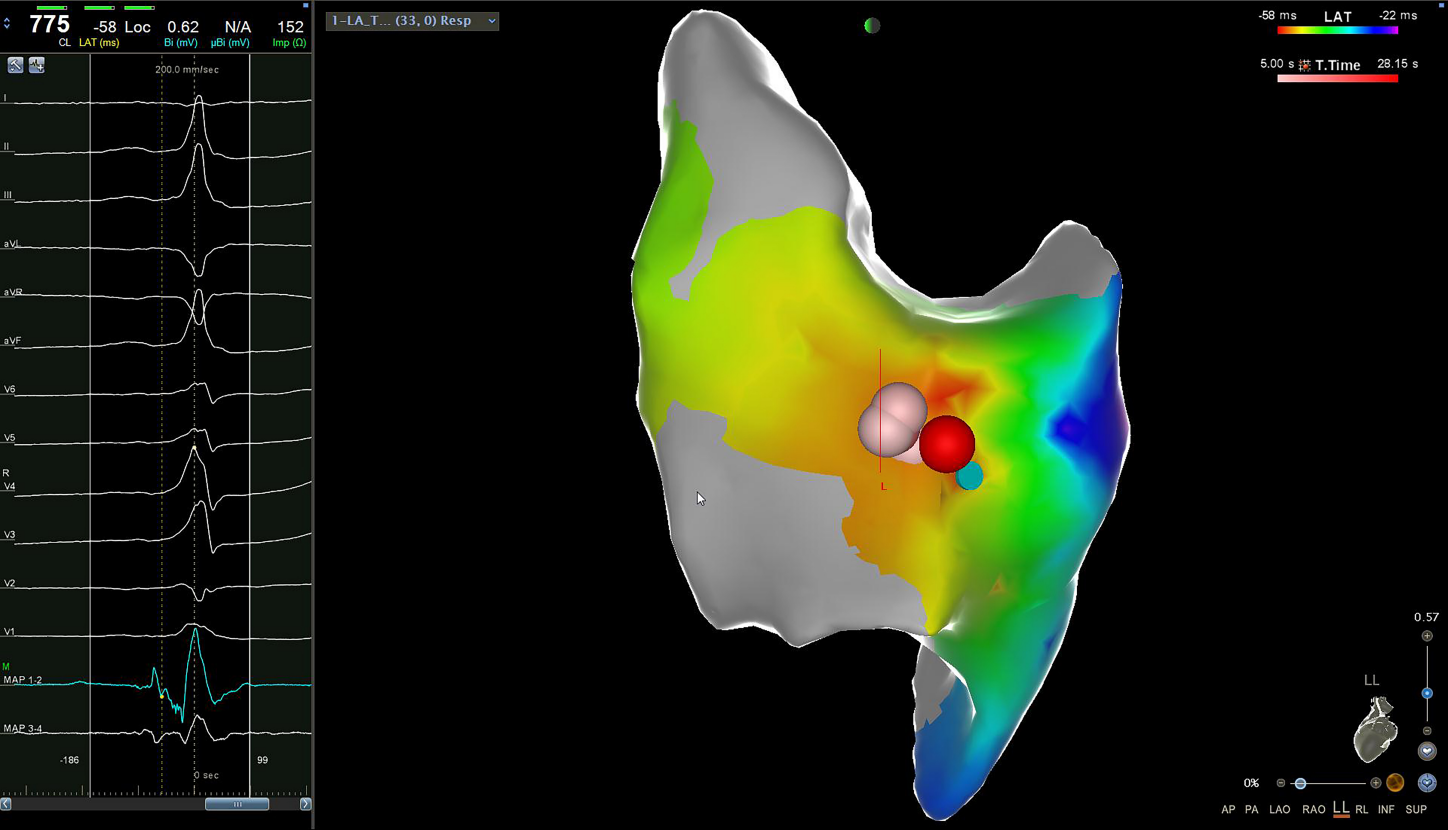
**A 3D mapping reconstruction was utilized to illustrate the left lateral localization of the accessory pathway (latero-lateral view)**. This 3D map, obtained with the CARTO system (Biosense Webster, Inc., Irvine, CA, USA), highlights the precise radiofrequency delivery locations. The red and pink dots on the map represent the targeted zone, with red dots indicating areas with a higher ablation index compared to the pink dots. 3D, three-dimensional.

A 40-year-old Asian man was admitted to our emergency department (ED) due to palpitations, atypical chest pain, diaphoresis, and dizziness that had arisen 36 hours before ED admission. Initially, managing clinicians were unable to retrieve medical history due to a language barrier. Later, a history of a multinodular thyroid goiter with hyperthyroidism and poor compliance to drug therapy with methimazole was discovered. Physical examination revealed a very fast heart rate (HR) with a 2/6 systolic cardiac murmur and diffuse enlargement of the thyroid gland. Blood pressure was low (around 90/50–60 mmHg). An electrocardiogram (ECG) (Fig. 1) showed mildly wide complex tachycardia with slurred QRS upstroke and slightly irregular cycle length. The initial differential diagnosis included antidromic atrioventricular re-entry tachycardia (AVRT), atypical atrioventricular (AV) node re-entry tachycardia (AVNRT) with bystander accessory pathway (RP not compatible with typical AVNRT), atrial tachycardia (AT) or atrial flutter conducted through an accessory pathway (possibly with 2:1 conduction), or theoretically SVT with aberrancy due to phase-3 bundle branch block. ECG morphology and Brugada criteria made ventricular tachycardia (VT) unlikely. An echocardiogram during tachycardia revealed an initial dilation of the left ventricle (LV) with overall normal LV function and secondary moderate mitral and tricuspid regurgitation. Due to hemodynamic instability, under conscious sedation, electrical cardioversion with 3 synchronized biphasic direct current shocks at 150/200/250 J was attempted. The first two shocks were incapable of restoring sinus rhythm; the third shock was able to interrupt the tachycardia for five beats, with a spontaneous new tachycardia initiation. While waiting for complete laboratory test results, ECG monitoring showed phases of irregular tachycardia cycle lengths (Fig. 2). Therefore, pre-excited AF was diagnosed lately. Using algorithms that can predict AP location, the ECGs suggested the presence of a left lateral AP. Subsequently, laboratory tests showed thyroid stimulating hormone (TSH) <0.001 mIU/mL and fT4 fairly beyond reference limits, consistent with a thyroid storm. Due to the unavailability of procainamide and/or ibutilide in the ED, an intravenous (i.v.) infusion of flecainide (150 mg) was started. At the same time, high doses of steroids and methimazole were initiated. Flecainide was stopped about three minutes after the infusion started due to a sudden acceleration of the tachycardia cycle length (about 200 ms), with the occurrence of wider QRS as well. A “watch-and-wait” strategy was attempted during the first hours in the ED, with continuous cardiac rhythm and invasive blood pressure monitoring, which were overall stable during the night. Six hours after the administration of methimazole and hydrocortisone, a spontaneous conversion to sinus rhythm occurred. Another ECG was collected, showing a short PR interval (90 msec) and a delta wave, more evident in the inferior leads (Fig. 3). During hospitalization, a gradual normalization of fT4 was witnessed; the LV showed inverse remodeling, and both mitral and tricuspid regurgitation ultimately normalized. Catheter ablation was initially considered, but the patient decided to defer it, despite physicians’ recommendations. Antiarrhythmic therapy with either flecainide or amiodarone was not started due to the risks of accelerating tachycardia and worsening thyroid function. A implantable loop recorder (ILR) was implanted to monitor arrhythmia recurrences. The patient was discharged 18 days after admission, with methimazole 5 mg bid, prednisone 25 mg, levothyroxine 25 mcg, and rabeprazole 20 mg. After discharge, the ILR monitoring showed only 2 episodes of self-limiting AF (HR 160/min, max duration 33 seconds), occurring 5 and 17 days after discharge, respectively. Sixty days after discharge, the electrophysiological (EP) study confirmed the presence of a left-lateral AP. Catheter ablation with radiofrequency was performed (Fig. 4). No complications occurred. After 19 months of follow-up with ILR monitoring, no arrhythmia recurrences were detected.

## 3. Discussion

### 3.1 Pre-Excited Atrial Fibrillation in the Emergency Department: Too often Unrecognized and Mistreated

WPW syndrome is characterized by specific ECG changes that lead to ventricular pre-excitation, known as the “WPW pattern”, and is associated with recurrent tachyarrhythmias [1]. The baseline ECG typically exhibits a short PR interval (<120 ms), a widened QRS complex, and a slurred upstroke (or downstroke) QRS, referred to as the “delta wave”. Ventricular pre-excitation occurs when an AP, a strand of myocardial cells with specific electrophysiological characteristics, activates both ventricles prematurely. The prevalence of WPW syndrome in the general population is estimated to be 1–3 in 1000 individuals [2]. WPW syndrome is associated with various SVTs, including AVRT and AF with rapid ventricular response, with AVRT being the most common arrhythmia linked to WPW, and AF occurring in up to 50% of patients with WPW [3, 4]. Several mechanisms may contribute to the development of AF in WPW syndrome. These include the spontaneous degeneration of AVRT into AF, the effects of the AP on atrial architecture, intrinsic atrial muscle vulnerability, and the role of autonomic tone. Pre-excited AF in WPW syndrome can potentially progress to VF, leading to syncope, cardiac arrest, and SCD. Factors such as syncope, a history of symptomatic tachycardia, younger age, multiple accessory pathways, accessory pathway effective refractory periods (APERP) <240 ms, and shortest pre-excited RR intervals (SPERRI) ≤250 ms are associated with an increased risk of developing malignant arrhythmias [5]. In this case, the electrophysiological study, with the use of isoprenaline, identified an APERP of 250 msec and a SPERRI of 220 msec. Given these considerations, accurate ECG interpretation is crucial, as misdiagnosing pre-excited AF could result in administering incorrect treatments that may induce VF [6]. Recognizing ventricular pre-excitation is particularly important in the ED, especially when dealing with wide-complex tachycardias or patients admitted for syncope with a potential arrhythmogenic cause [7]. Various arrhythmias, such as polymorphic ventricular tachycardia and fast AF with aberrant conduction due to bundle branch block, can mimic pre-excited AF [8, 9]. Even in cases of particularly fast pre-excited AF, where the tachycardia cycle length is not notably irregular, AVNRT with aberrancy should be considered [10]. Despite the importance of recognizing signs of pre-excitation in WPW, pre-excited AF is often misdiagnosed, with ventricular tachycardia being the most likely incorrect diagnosis [11]. A study by Koźluk *et al*. [12] found that members of ED medical teams have limited skills in recognizing WPW syndrome with rapid AF, resulting in correct treatment in only 15% of cases.

### 3.2 Pharmacological Treatment of Pre-Excited Atrial Fibrillation: Evidence and Pitfalls

In the acute setting, according to current guidelines [1], the pharmacological treatment of pre-excited AF in stable patients should avoid AV node blocking agents (particularly adenosine) since they may facilitate a potential induction of fast AF [13]. The preferred administration of intravenous drugs such as Ibutilide or Procainamide (Class IIa, level of evidence A), or class IC drugs like Flecainide or Propafenone (Class IIb, level of evidence B) is recommended in this scenario [1]. However, it is essential to note that IC drug agents are not entirely risk-free as they exert an effect on the AV node [14, 15]. Flecainide, a sodium channel-blocking drug, decreases the rise rate of phase 0 (resulting in depressed conduction velocity and prolonged conduction time), with little effect on the action potential duration, and it prolongs atrial and AP effective refractory period. Previous studies have documented that class I antiarrhythmic drugs could exhibit a proarrhythmic effect in various settings, mainly accelerating the ventricular response of supraventricular tachycardia [16]. Indeed, both the lack of a significant effect of IC drugs on the AV node and the effect on atrial and AP conduction could lead, at least in some cases, to an acceleration of ventricular rate response. In the chronic setting, amiodarone is no longer recommended (Class III) as it may often enhance AP conduction, with subsequent reports of ventricular fibrillation development [17, 18].

Synchronized direct current (DC) cardioversion is indicated (Class I) in hemodynamically unstable patients, as in our case where the patient presented with low blood pressure due to the long duration of a high ventricular rate tachycardia, or whenever drug therapy fails to convert or control the tachycardia. Patients with pre-excited AF may exhibit a poor response to antiarrhythmic drugs due to their weak action on AP conduction and no significant inhibition of AF recurrences. Furthermore, electrical cardioversion can only temporarily terminate the tachycardia with no real suppression of the recurrence of AF. In such cases, it is important to evaluate all possible concomitant factors that could play a role in perpetuating arrhythmogenesis, such as heart failure, infections, organic heart diseases, and hormonal imbalances, such as thyroid storms.

### 3.3 Treating the Underlying Causes: After a Storm (Often) Comes a Calm 

Thyroid storm is an endocrine emergency with a mortality rate of up to 10–30%, necessitating prompt recognition for timely initiation of treatment [19]. Individuals with hyperthyroidism face an increased risk of cardiac arrhythmias, particularly AF, with a prevalence of 5 to 15% [20]. While the association between new-onset AF and hyperthyroidism is uncommon in the absence of additional signs and symptoms of hyperthyroidism, as reported by Klein *et al*. [21], up to 13% of patients with unexplained AF may exhibit biochemical evidence of hyperthyroidism, highlighting the importance of measuring serum thyrotropin in patients with new-onset AF [21, 22]. The treatment of hyperthyroidism typically involves intravenous steroids and methimazole, as seen in our case, along with β-blockers. The excess of thyroid hormone can act as a trigger for the onset and perpetuation of tachyarrhythmias, necessitating the use of β-blockers. Managing pre-excited AF during a thyroid storm poses challenges due to the need to avoid β-blockers in this scenario. In a similar case described by Naqvi *et al*. [23], acute management involved amiodarone, metoprolol, steroids, and methimazole, with amiodarone chosen due to a national shortage of procainamide. While these authors considered amiodarone’s side effects on the thyroid gland negligible given the prompt effectiveness of methimazole and prednisone, caution is warranted. We do not recommend using amiodarone in this setting, not only due to potential additional risks on thyroid function but also because of enhanced accessory pathway conduction, potentially leading to ventricular fibrillation, as previously mentioned and reported in European Guidelines [1].

Treating hyperthyroidism has been reported to result in spontaneous cardioversion in nearly two-thirds of patients [20]. In this case, considering the waiting time between steroid and methimazole administration, we believe this was also the case. The mortality in patients with pre-excited AF due to thyroid storm is unknown. If catheter ablation remains the treatment of choice for symptomatic pre-excited AF (Class Ib) [1], an electrophysiology study should be deferred in this particular scenario until the complete resolution of thyroid storm. To maintain sinus rhythm, patients might be started on antiarrhythmic drugs (AAD) until catheter ablation. However, due to the pitfalls of amiodarone and potential risks associated with accelerating AF recurrences or converting it into atrial flutter with 1:1 conduction when administering flecainide, AADs were not initiated in this case. Moreover, these episodes were ascribed to the thyroid storm in a WPW syndrome with the accessory pathway likely being silent in the patient’s previous medical history. Nevertheless, the decision was made to monitor recurrences with an ILR for thorough monitoring, allowing consideration of eventual AAD therapy in case of arrhythmic recurrences, balancing the risk-benefit ratio. Proper hyperthyroidism management significantly helped the patient overcome AF recurrences, and they were safely managed until catheter ablation when they decided to undergo the procedure.

### 3.4 Catheter Ablation in WPW Syndrome: Is the War Over? 

Catheter ablation of an AP has a high success rate (95%) and is associated with a low complication rate (3%), which mainly varies based on the AP location and the operators’ experience [3, 4]. Major complications include cardiac tamponade (0.13–1.1%) and complete AV block (0.17–2.7%), primarily observed in patients undergoing ablation of a septal/parahisian AP [24]. During the EP study, proper localization and subsequent catheter ablation of the AP are usually performed in sinus rhythm (for patients with an overt AP) or during ventricular pacing (for patients with a concealed AP). However, the procedure may be complicated by the occurrence of AF. Several studies have reported cases of AP catheter ablation performed during pre-excited AF, developed during the procedure [25], although not specifically during a thyroid storm. Kose *et al*. [26] were the first to report successful mapping and ablation of an AP in two patients during pre-excited AF, later confirming their findings in an eight-patient cohort. Previously, Hindricks *et al*. [27] reported successful localization and ablation of AP during AF in 18 of 19 patients with left-sided APs and in 2 patients with right-sided Aps [26, 27].

Based on these encouraging data and considering the need for an invasive strategy in patients presenting with unstable, refractory, and/or recurrent pre-excited AF, Chen *et al*. [28] reported the results of five patients with high-risk pre-excited AF who underwent emergency catheter ablation of the AP to effectively correct the hemodynamic instability induced by the unresponsive rapid conduction of AP in pre-excited AF. No complications were observed or reported in this study, but it’s important to note that no patients with thyroid storm, which poses a very high risk of recurrences, were included. Therefore, in patients presenting with recurrent pre-excited AF episodes refractory to acute electrical cardioversion and/or intravenous antiarrhythmic therapy, catheter ablation of the AP might be considered an emergency procedure to reduce the risk of life-threatening arrhythmias and sudden cardiac death, once it is established that the underlying cause must be treated as well.

## 4. Conclusions

Pre-excited AF with rapid ventricular response poses a challenging clinical scenario. Given its potential evolution towards life-threatening events, prompt electrocardiographic recognition is essential for appropriate management. It is crucial to develop new educational strategies aimed at increasing the skills of healthcare workers in the ED to recognize this arrhythmia effectively. Once an appropriate diagnosis is made, evaluating, and treating all concomitant underlying factors potentially playing a role in perpetuating arrhythmogenesis, such as thyroid storms, is imperative. In challenging cases involving hemodynamically unstable pre-excited AF that is refractory to antiarrhythmic drug therapy and cardioversion, emergency catheter ablation could be a reasonable treatment option.
